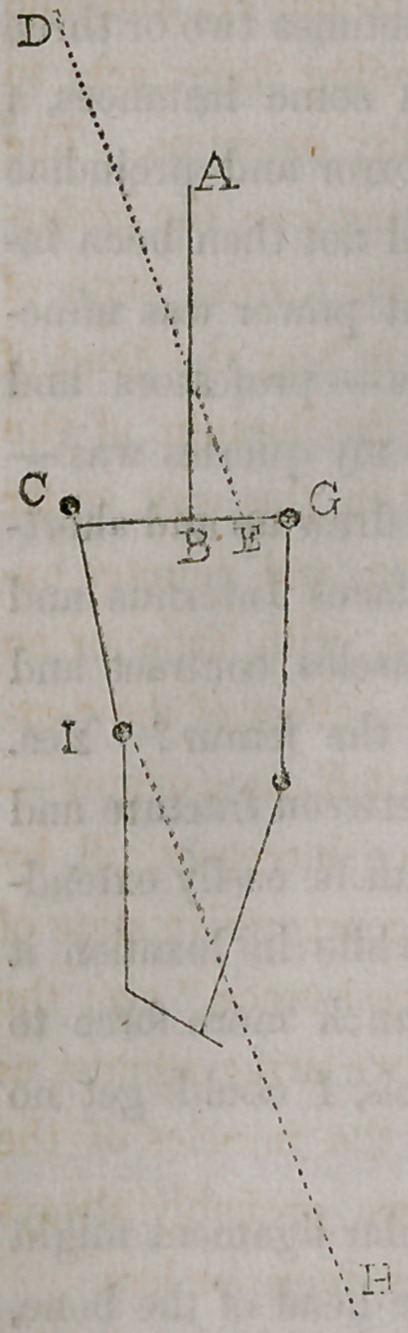# Dislocation of the Femur on the Dorsum Ilii, Reducible without Pulleys, or Any Other Mechanical Power. Three Cases

**Published:** 1851-08

**Authors:** W. W. Reid


### BUFFALO MEDICAL JOURNAL

AND

### MONTHLY REVIEW.

VOL. 7. AUGUST, 1851. NO. 3.

### ORIGINAL COMMUNICATIONS.

ART. I.—*Dislocation of the Femur on the Dorsum Ilii, reducible without Pulleys, or any other Mechanical Power. Three Cases.* An Essay read before the Monroe County Medical Society, at its Annual Meeting, in the City of Rochester, on the 8th May, 1850. By W. W. REID, M. D.

DR. FLINT,

Dear Sir:—The following paper was read before the Monroe Co. Med. Society, at its Annual Meeting, in the City of Rochester, on the 8th of May, 1850. For reasons, which are obvious, and *some, which are not so obvious,* I am induced to offer it for publication in your Journal. If you deem it admissible, and worthy, will you give it an insertion in your next number.

Respectfully and truly yours,

W. W. REID.

Rochester, June 24th, 1851.

GENTLEMEN,

I propose to show that Dislocation of the Femur on the Dorsum Ilii, may be reduced without pullies, without Jarvis' adjuster, without Fahnestock's twisted ropes, without an assistant, in less time, and with far less pain, than by any mechanical means whatever, simply by the hand and strength of the operator alone.

The announcement of a proposition so novel, so ultra, and contradictory to the teachings of all standard writers on surgery for the last hundred years, exposes me, I am aware, to the sneers of some, to the pity of others, and to the charge of rashness, by all, and requires that I make good my statement by undoubted and substantial proof.

The subject matter of this paper has been written, but withheld from the public and profession, several years, principally for two reasons:

*First.*—The theory and practice here recommended are so diametrically opposed to all our highest surgical authorities, whether among the living or the dead, that I have shrunk from the obloquy and opprobium that are apt to attach to an innovator upon long established opinions, dogmas, and practices, especially when held and taught by men in our profession of profound science, and practical skill.

*Second.*—I had to wait some four or five years for an opportunity to put to the test this mode of reducing a luxation of the hip joint, before a case presented itself in my own practice. In the spring of 1844, the first opportunity offered, but as "one swallow does not make a summer," I was still unwilling to venture before the profession, although so far as one case could establish a principle, this one did so, as we shall see directly. During the past year, (1849,) two other cases have fallen into my hands, and have rendered what was before certain to my own mind, "doubly sure."

As the facts and views here adduced call in question, and entirely controvert several important dogmas of Physiology and Surgery, taught as *truths*, by the Bells, Sir A. Cooper, S. Cooper, Ferguson, Druit, Liston, Chelius, South, Physic, Wistar, Dorsey, Mott, Warren, Gibson and other eminent teachers of Surgery, I may be pardoned, if I briefly sketch the mental process, the observations and experiments by which I arrived at conclusions so diverse from the teachings and experience of such eminent surgeons.

During the years 1826, 7, and 8, while a student of medicine and surgery, it was my fortune to witness several cases of luxations of the head, and fractures of the neck of the femur. We had at that time in our embryo city of Rochester, of ten thousand inhabitants, a corps of some six surgeons and physicians of as great efficiency and skill as any town of its size could boast. When so important an operation as the reduction of a hip joint was to be performed, several, if not all of these gentlemen, usually met, together with their students, and among them, myself.

Having witnessed, on several occasions, the *inquisitorial torture* inflicted upon the unfortunate patients—their screeching—their piteous begging to be released — the slipping of bandages — the yielding and re-adjusting of fixtures — the delay — the duration of the operation, sometimes two or three hours — the exhaustion of the patient, and after all, in some instances, a failure, and the patient a cripple for life, a profound horror and prejudice against the use of pulleys seized me, (Jarvis' Adjuster had not then been invented) and I could not avoid the conviction that a great power was unnecessary, and that it must be misapplied. Preceptors — professors and authors were interrogated — the unanimous reply to all my queries was — "to overcome the contraction of the great muscles, which drew up and shortened the limb, viz. the Glutei, Triceps Femoris, the Iliacus Internus and Psoas Magnus." But do not these same powerful muscles contract and shorten the limb when there is fracture in the neck of the femur? Yes. And you tell me that one of the diagnostic symptoms between fracture and dislocation on the dorsum, is, that in fracture the limb can be easily extended to its normal length, by the strength of one man, while in luxation it cannot. Now why do these great muscles require so much more force to overcome them in one case than in the other? To this, I could get no satisfactory, nor even a plausible reply.

The next reflection that arose, was, perhaps the capsular ligament might be merely rent by a slit, so as to permit the escape of the head of the bone, and thus grasp it around the neck, and consequently, when forcible extension was made on the limb, the ligament must be torn up to admit the return of the head, to the acetabulum. But Sir A. Cooper says no; for he had dissected two or three dislocated hip joints, and always found the capsular ligament completely torn up, so that it could offer no resistance to the returning bone. This, however, is but negative proof, and might not apply to all other cases that have occurred the world over, and which he did not dissect — nor does it appear but that in those he did dissect, the ligaments had been torn up by the application of pulleys — and not by the force that dislocated the bone. It is not doubted or denied, that in some instances the ligaments are completely broken up, by the dislocation; but admitting that Sir A. Cooper and his followers are right, then there must still be a reason for the difference of power required between a luxation and a fracture to extend the limb to its normal length. It may be in the impracticability of the instrument; for it is evident, on the slightest inspection, that the action of the pulley is *indirect*, most awkward, and unscientific in a mechanical point of view. This is easily illustrated by a simple diagram. Let A. B. represent the axis of the body; C. G. the transverse axis of the pelvis; C. I. the dislocated femur; D. E. the counter extension; I. H. the extension and direction of the form by the pulley. N. B. The positions here given are those directed by the most approved writers on surgery. Counter extension is here represented as being made from the perineum of the side opposite the injured limb; for, as professor Gibson and others very justly remark, counter extension on the perineum of the injured side greatly irritates the abductor muscles, stimulates them to contract, and thereby confines the bone and prevents its mounting over the edge of the acetabulum, defeating the very end we are striving to attain. But *mechanically*, it is the very worst point, or position, that can be selected, hence authors do not agree in their directions. *Physiologically* it is the best, but *mechanically* it is the worst. Let us refer to our diagram. D. E. being the line of counter extension, E. becomes a kind of fixed point, and as it were a center, about which we describe a circle, whenever we apply force, by the pulley in the direction of the line I. H. For it is evident, that force thus applied, has a tendency to bring all the movable points into one and the same straight line, with the two opposing forces — that is, to bring the points E. C. and I. into a direct line with D. and H. Consequently, the points A. and C. move in a circle around E. which, in reference to A. and C. is a fixed point, yet it moves laterally in a right line towards C. till it comes in a right line with D. and H. Now the effect of this, is to *abduct powerfully*, the dislocated femur, and thereby "irritate the abductor muscles and stimulate them to contract, &c.; and thus, by this *indirect* action of the pulley, we defeat our own intentions — cruelly torture the patient, and perchance fracture the neck of the bone — an accident, that has occurred more than once, in the hands, too, of eminent surgeons, both in this country and Europe. And, as if to render this accident more certain, these same men, and others renowned for scientific attainments, have recommended a practice, which, to say the least of it, manifested the most deplorable ignorance of the science of mechanics — I mean, the placing of a strap or similar appliance, under the thigh, close to the pelvis, and then attempting to lift it into the socket, while extension is being made — some have even applied another pulley laterally. In my judgment there is no reprehension too severe for such a practice, and the professor who would teach it, should be turned back to a class of sophomores o study *mechanics*, especially the power of the compound pulley.

But suppose we change our counter extension from the sound to the injured side, the point E. is nearer to a right line with D. and H., and the lever of C. E. is shortened, and consequently the extending force acts so much more directly; but then another and worse difficulty meets us. The counter extension band on the perineum, passes over and confines the *abductor* and rotator muscles, all of which are already in their utmost state of tension, and the moment force is applied they are made to hug the head of the bone, if possible, still more immovably, down upon the dorsum of the ilium, behind the brim or ridge of the *acetabulum;* and in this way, so far as these muscles are concerned, our forces, both extension and counter extension, are expanded upon these muscles themselves, with little or no tendency to reduce the bone. While here, as before, the effect of the " extension, is to rotate the body and pelvis around the point E. as a center — *abducting* the fractured thigh more and more as the force increases, till, by and by, we bring other muscles, which have been in a comparative state of rest — or very partial tension, into violent *tension* and resistance," viz: the *iliacus internus*, *psoas magnus*, and *triceps* — and thus we array against us (unnecessarily as I shall show) the power of nearly all the muscles of the joint. And, as I shall have occasion to note hereafter, we probably always rupture the *pyriformis*, and indeed several other muscles, more or less.

These remarks and observations were originally made in reference to the use of the pulley, as "Jarvis' Adjuster" was not then known to me, but they will be found to apply, in a great measure, to the action of the latter also. But it must be admitted that it is less objectionable than the pulley, for reducing dislocations, while it has many other valuable uses, to which the pulley cannot be applied, but for dislocation of the hip it is entirely unnecessary.

For the first ten years of my professional life, the subject of dislocated hip on the *dorsum ilii*, was never long absent from my thoughts. Its investigation was repeatedly laid aside and taken up whenever anything occurred to recall it. One day, while sitting with the skeleton before me — the femur dislocated, and the head held firmly with one hand, traction and evolutions being made with the other — studying the relative condition and action of the muscles, and observing how severely some of the abductors and rotators must be treated, it suddenly occurred to me, that it would be important to know how much they would *elongate* beyond the normal length before they would rupture. "*To tire out*" and "*stretch*" muscles, was a common expression of authors, when advocating the use of pulleys. But whether they intended by such language merely to convey the idea of overcoming the contraction of a muscle, when *shortened* by its *natural* action as when its origin and insertion had been approximated, as in dislocations, or whether it was meant to extend the muscle beyond its *normal* length, I could not ascertain — both ideas seemed to be entertained. I determined to settle the question by actual experiment. That a *contracted* or *shortened* muscle could be "tired out," and "stretched" to its normal length, was evident enough — but how much further could it be extended without rupture? And what power was necessary to thus extend it? These were the questions I proposed to myself.

I procured the fore-leg of a sheep at the market; said to belong to an animal two years old and two days killed. I dissected up and separated, from all its fellows, one of the *flexors* — a ribbon like muscle, seven inches long, and one-eighth inches wide, and three-sixteenths inches thick — a small and elegant muscle. I left it attached to the bone at its origin, but cut off the tendon at its insertion, and wound it with fine iron wire, making a loop by which to suspend weights. Before applying any weights, the fibres had a wrinkled or puckered appearance. I marked two points, one at its origin, the other at the upper coil of the wire wound around the tendon — the distance between them five inches. I then suspended a two ounce weight in the loop of wire; the muscle *immediately elongated* a quarter of an inch — the fibres became straight and smooth; I then added one pound, no elongation; then two pounds, length the same; then four pounds, no change; then seven pounds, no alteration. Thus I continued to add weights and then measure, till I had suspended fifty-seven pounds to this small muscle, and not the least perceptible alteration in length could I discover after the first two ounces, (which were sufficient to 'tire it out,') till I added the fifty-eighth pound, when it suddenly tore in two, and the weights came to the floor. One half of the fibres first yielded at the lower end, where the wire grasped the tendon. On inspection, it appeared that I had wound the wire so high as to embrace a few of the fleshy fibres; these first gave way, while at the upper end of the muscle the other and opposite half of the muscle broke, and thus it split in the middle, its whole length. This result surprised me. Here was a muscle, slender, isolated, deprived of all support by its aponeusoses, and connections of cellular membrane to its fellows — belonging to a young animal, not remarkable for its strength of muscle, and without *vitality*, *supporting* fifty-seven pounds, without the least perceptible elongation beyond its normal length. How much power then would *all the large living muscles* of the hip joint of a strong man, require, to elongate them even one-eighth of an inch?

Wishing to determine how much support the fascia and cellular attachments would add to its power of resistance, I prepared a similar muscle, leaving it entire, but cutting off all the other muscles and ligaments. In other words, I divided the leg through the knee joint, and left one muscle undivided. I suspended it as before, attaching the weights to the leg, below the insertion of the muscle to be extended. But this broke with forty-seven pounds. I attributed this to the oblique action of the weights — it being very difficult to adjust the suspended bone covered with flesh so as to keep all the parts in a direct line.

In my next experiment, I dissected up all the tendons of the muscles about the knee joint, without dividing them, but divided all the ligaments, thus opening the joint. The muscles and facia were all left in their natural state. The skin was removed of course before I obtained the leg, but in all respects was similar to the others.

Before weights were suspended to it, the ends of the bones were in close contact in the joint, and would not admit the introduction of the point of a pen-knife blade. The weights were added by degrees, the ends of the bones carefully noted and an attempt made, from time to time, to pass the point of a pen-knife blade between them — but this could not be done till 200lbs. had been added. When a few pounds had been applied the limb began to come into a right line. The ends of the bones on the front side of the joint, that is, on the side of the extensors, were more firmly pressed together. As the weight was increased, the tendons of the *flexors* became very much strained, while those of the *extensors* became quite slack. Hence thus far in the experiment, the whole weight was sustained by the *flexor* muscles, owing to the fact that the extensors have a greater normal or comparative length than the flexors. With a weight of 300lbs. the bones began to separate, so as to admit the point of a pen-knife. A portion of the weight was then removed, when the bones at the joint returned and came in contact again, which seemed to prove that the muscles had elasticity and were capable of some elongation without rupture. The weights removed were re-applied, and forty pounds more added — when the bones separated about one-eighth of an inch. A portion was again removed, but the bones did not return readily, nor closely — the joint seemed loose. They were then carefully re-applied, when the flexor muscles yielded, suddenly throwing the whole weight on the extensors, which broke at once, seeming to offer but little resistance. Thus it appears that the flexors sustained the whole 340lbs. which the extensors were not able to do — and that the flexors were incapable of extension or elongation, very little over *one-eighth of an inch* beyond their natural length, without rupture.

It was my intention to have pursued and varied these experiments, so as to have established or refuted the conclusions to which they seemed to point, and which have since become the convictions of infallible truth in my own mind, however defective the proof and illogical the process of reasoning. But professional labors and interruptions have conspired to prevent their prosecution, and I shall leave them to be pursued and perfected by others who have more time and zeal for prosecuting such investigations.

After making the above experiments I was convinced that I had discovered the real difficulties to be overcome in reducing a dislocation of the hip on the *dorsum ilii*, viz. the extension to their utmost, or nearly so, of the *obturator externus, and internus*, *quadratus*, *gemini*, *pyriformis*, and *pectineus*, — and their incapability of but little more extension — and that all traction downward, on the fractured limb, only increased this tension, and could do nothing towards bringing the bone into place, except at the hazard of almost certain rupture of some of these muscles, and of a fracture of the neck of the bone.

I now recommenced my manipulations and evolutions on the skeleton, to ascertain how this indirect, and not merely useless, but absolutely detrimental action of the pelvis could be avoided. It was soon obvious that these muscles, instead of being extended further, could all be *relaxed*, and their natural *action* and *contraction* be made to draw the head of the bone back into its socket, and that instead of employing all *our power to overcome them*, we could actually use all *their power to aid us* and do the very work for which we were in vain employing the compound pully, at an immense disadvantage. And all this is done by simply carrying the injured femur in the only direction in which, in fact, it can be moved, viz. inward and over the sound one, and upward and over the abdomen, flexing it upon the pelvis till the knee is carried up as high as the *umbilicus*, and outward on a line with the same or injured side — then turning the toe outward — the heel inward — the foot across the opposite and sound limb, and carrying the knee outward and downward, and making gentle rotations of the thigh — when the head slips in easily, with a slight jerk, an audible snap — and the whole limb slided down easily and gently into its natural position beside the other. The whole operation can be performed easier, and in less time, than it can be described.

The conviction was so strong in my mind that this method was certain and practicable, that I no more doubted it then than I do now, after having demonstrated it in three several instances, two of which were within the last year. And so impatient was I to put my theory to the test, that I believe I almost wished every day (wickedly perhaps) that some one would dislocate his hip, and give me an opportunity to reduce it.

I was aware that Professor Nathan Smith, of New Haven, had, in his day, taught in his lectures, a somewhat similar method, — perhaps the same; but none of his pupils, whom I had ever met, could describe either his method or the rationale of it. I had seen, too, his memoirs, published by his son, Professor N. R. Smith, of Baltimore, but he confesses that he did not recollect the teachings of his own father, and that he, the elder Smith, had left no notes or records of his doctrines or practice. Dr. N. R. Smith, however, proceeds to give what seems to him the probable doctrines inculcated by his father, and gives directions for reducing dislocations of the hip, with drawings illustrative of his method. But it is apparent, that, when he wrote his book and gave these directions and illustrations, he had never reduced a hip by his method. For his directions require impossibilities, and his illustrations are mere fancy; no such thing in nature can exist. For to *abduct* a thigh dislocated on the *dorsum* of the *ilium*, before flexing it on the pelvis, or to *abduct* and *flex* at the same time, as he directs, is absolutely impossible, without rupturing the obturator externus — and to rupture this, in order to obtain flexure, would require the power of many men; *but to flex the leg first on the thigh — then adduct the thigh, carrying it even over the sound one and at the same time, flex the thigh on the pelvis, carrying the knee over and upward by a kind of semi-circular sweep*, is a very different and a very easy thing.

Case 1. — In the spring of 1844 — [I give this case from recollection, the notes which I made of it having been mislaid] — I was called to see a strong, robust Irish woman, of whom they gave me the following history: Four days previous, while out at washing, about three-quarters of a mile from her own residence, she slipped and fell down a flight of steps — could not rise — and when helped up, could not stand. She made a great out-cry but as no blood was visible, she was thought to make a great "fuss for nothing." Her husband, who was an intemperate carman, was sent for. He put her on his cart, drove her home three-quarters of a mile; when he arrived there, not being able to lift her, he dumped her down at the gate as he would a load of dirt. The neighboring women helped him carry her in, and place her in bed. For four days they assiduously fomented her hip, of which she complained greatly; but it swelled considerably and became "black and blue." They now began to think the woman was "*hurted.*"

In this condition I found her. A single glance at the position of the knee and toe, created a strong suspicion of dislocation, but an attempt to *abduct* and rotate the limb, gave great pain and determined the nature of the accident. Although the patient was suffering considerably, I was in ecstacies, and felt really obliged to her, not so much, I hope, for dislocating her hip, as for the opportunity she afforded me to reduce it. I called in Doctors M. Strong and the elder Bradley, and Mr. now Dr. Hammond, to assist me. I stated to them my determination to reduce it, if possible, without the use of pulleys, and explained my method. Nevertheless I had provided myself with compound pulleys, to be used in case of a failure. As the accident was of four days standing, the hip considerably swollen and inflamed, and the patient quite muscular, I took the precaution to bleed her freely, and give her tart-antimony till nausea was produced. She was in the meantime placed on a lounge, on which a wide board was laid and covered with a folded quilt. This made a firm table about fourteen inches high, and about twenty inches wide, which gave me the opportunity of throwing the whole weight of my body on the flexed limb, if I wished, while it gave me perfect command and control over it in every way. The patient was placed on her back, and a sheet folded lengthwise thrown across the upper edges of the pelvis bones, and each end given to an assistant, for the purpose of fixing the pelvis. Placing myself on the right and injured side, I seized the knee with my left hand, and the ankle with my right; I then flexed the leg upon the thigh; at the same time, slowly carried the knee and dislocated femur, over the sound one, pressing it firmly down upon it — and upward over the pelvis, constantly pressing it close to the body, moving it upward with a circular sweep over the abdomen, till the thigh was in a line with the right side of the body and the knee, pointing towards the right axilla. While the thigh was being carried up to this position, the bone or axis of the femur, was performing a kind of rotation on itself, whereby the toe was coming more outward and the heel more inward. In other words, as the knee went upward, the *obturator externus*, *quadratus*, &c., drew the head of the bone downward, and inward towards its socket. When the knee and thigh were in the position above indicated, the heel was strongly rotated inward, the knee drawn outward, and the foot carried across the thigh of the sound side, when the head slipped into its place, and the limb glided gently down into its natural position. In doing all this, comparatively very little force was employed, and very little pain produced, for the obvious reason, that, by this evolution, the muscles that were in a state of extreme tension and irritation by the displaced bone, were gradually relieved and relaxed, as the head of the bone descended and approximated its proper place, which it did by the action of these same extended muscles.

It will be perceived, that by this mode of operating, we make a *lever* of the shaft or bone of the femur, and a fulcrum of the edge of the pelvis — and by this means lift or dislodge the head of the bone, — while the abductor muscles draw it downward and inward, making it, as it were, *back into* its place, through the rent of the capsular ligament. Whereas, if it were drawn by direct force, as by the pulley, the head and neck of the bone would act as a kind of hook, and would tear away the capsular ligament, if it were only slit, and as I believe it often, if not always, does tear off the tendon of the *pyriformis*, as I shall endeavor to show presently; for the *abductor* muscles are so strained, and hold the head of the bone so firmly to the dorsum, behind the ridge of the *acetabulum*, that it is next to impossible for it to mount over this ridge and into the socket, and must therefore descend behind it tearing every thing before it — ligaments, muscles and all — and hence the immense power required to reduce it by these means, and hence, too, the failures, the fractures of the neck, and the cripples, that have been made for life, by this barbarous and unscientific mode of reduction.

Case 2. — On the 31st of July, 1849, Mrs. Cornelius Christie, aged about 38 years, was thrown from the top of a load of household furniture, with a small child in her arms. Mother-like, she held fast to the child, which received no harm; but, falling among and upon the furniture, she had the perineum and vulva considerably lacerated, and her right hip dislocated. I saw her within one hour after the accident. Doctors Bowen, Brown, and Holton, were in attendance when I arrived in company with Dr. E. P. Langworthy. The patient was placed at once in the position as already described in case No. 1, when I proceeded, in like manner, to operate; but the wound in the perineum and vulva occasioning great pain, on the attempt to flex the thigh, I desisted, and gave a full dose of morphine — not having any chloroform on hand. We waited three-fourths of an hour for the effect of the morphine. I then, as already described, seized the knee with one hand — the ankle with the other — flexed the leg on the thigh — the thigh on the pelvis, carrying it *inward and over the sound limb* — then upward over the abdomen, till the thigh was nearly parallel with the right side — then rotated the heel inward, carried the foot over the sound thigh, and the knee outward, when by a gentle oscillation and rotation of the thigh, the head slipped into the socket. The whole time required in this operation did not exceed *two minutes*. The force employed, and the pain suffered, were too trifling to be named.

Case 3.— On the 2d of Dec., 1849, early in the morning, I met Dr. E M. Moore, Prof. of Surgery in the Woodstock and Berkshire schools of medicine. He informed me he had been called up in the night to attend a case of dislocated hip. I jestingly said, " I wish you would let me show you how to reduce it." He replied as jocosely, " I understand you have got some new-fangled notions about dislocations, and I should like to see you try your skill." He desired me to explain my method. I did so, illustrating it by manipulations on the skeleton in his office. He agreed that I should make the attempt; but, that the full merit of my mode of operating should be brought out, he proposed that I should have no aid from any of the usual adjuvants, such as the warm bath, nauseating doses of antimony, bleeding, opium, nor chloroform. To all this I consented.

The patient, William Fagan, was a strong muscular Irishman, 52 years of age. He was placed on a lounge, on a board covered with a folded blanket, as already described — two assistants, one on each side, steadied the pelvis. I proceeded in all respects as above stated in the two preceding cases, and in about *two or three minutes* reduced the dislocation. Doctors Moore and Cruttenden, Mr. D. Bly, and other students of Dr. M. were present.

To those who have never witnessed this mode of operating, these statements may seem incredible, yet so simple, easy and short is it, that Dr. Moore declared, that " hereafter any fool might reduce dislocation of the hip on the *dorsum ilii*." Although in the three cases given above, I used a low table, yet I believe the floor is better, and all that is necessary. I used, too, a folded sheet thrown over the pelvis, and had it held down on each side by an assistant; but even this is unnecessary, and is, moreover, always in the way, after the thigh has been flexed to a right angle with the spine or axis of the body; when the thigh has reached this position we have perfect control of the pelvis, and can fix it firmly, by pressing the thigh strongly down upon it. So simple, too, is the operation, that if the patient be a female, and it were required to reduce the joint without exposing the person, it can be done, under a light covering, or under even her own dress, if sufficiently loosened.

On the 18th of December, just after the occurrence of the third case above narrated, Dr. Moore had a subject in process of dissection by his students, when he proposed to me that we dissect up the muscles of the hip joints, leaving them *in situ;* dislocate the bones, and then operate on them by traction in the usual way, and also by flexion after my method, in order that we might observe the condition and action of the muscles, before and during both modes of operation. We found it impossible by the power of our hands alone to force the head of the bone through the capsular ligament, till we made a slight incision into it. The head then shot through it, tearing it sufficiently to permit its passage, but then the ligament seemed to fit close around the neck of the bone. As the head passed out backward and upward, it caught the tendon of the pyriformis, *tearing it off as it passed underneath and above it,* which, if it had remained entire, would have brought its tendon, like a cord, across the neck close to the head, lashing it firmly down to the dorsum of the ilium. We were at the time inclined to attribute its rupture rather to the decayed state of the subject, than to excessive distension by the dislocation. But precisely the same thing occurred in dislocating the other hip. It is true this muscle was also in the same *stale* state; and the accident may, perhaps, have happened in both instances from the like cause.

When dislocated, the head of the bone rested on the *gluteus minimus* muscle! The *gluteus medius* and *maximus* were shortened and relaxed—so also were the *iliacus internus*, *psoas magnus*, *adductor triceps* and *pectineus.* Till now I had supposed that this last named muscle would have been among those that were put upon the stretch. Posteriorly the *obturator internus*, *gemelli* and *quadratus* were greatly strained; and it was apparent, that the *pyriformis,* if it had not been torn off, would have been even more so. Anteriorly, the *obturator externus* was stretched, seemingly, to its utmost, *adducting* the bone powerfully. It is this powerful muscle, which so firmly fixes the limb; turns the toe and knee inward; prevents rotation and abduction, and gives such excruciating pain to the patient when any such attempts are made.

Here, then, are two sets of muscles, acting in direct antagonism to each other, and both strained to their utmost tension. One set, drawing the bone backward and rotating it *outward.* The other, *adducting* and rotating it *inward.* Some might be inclined to puzzle themselves to know how these two sets of muscles, one situated before and the other behind, could both be in a state of tension, when the bone is thrown backward toward and in the direction of the latter. The explanation is very easy. Although the head of the bone is thrown backward, yet the great *trochanter* and shaft of the bone is thrown forward and rotated inward. So that the *pyriformis*, *obturator internus*, &c., which are inserted at the root of the *trochanter*, are necessarily elongated, while the anterior *obturator externus* runs backward behind and around the bone, to be inserted at the root of the trochanter, in order to rotate the limb outward it must also be strained just in proportion as the limb is rolled inward, and the trochanter is carried upward The *quadratus*

is stretched for the same reason, viz., its point of insertion is carried upward and inward.

After having carefully noted the relative position of the bone and muscles, we made traction on the femur, downward and inward, over the sound limb, as we are directed by the most approved authors, but the moment the attempt was made, the muscles already named as being in a state of tension, became more tense, and bound the head of the bone more firmly down on the *dorsum;* and although all the muscles about the joint were separated from each other — were loose, without vitality and almost in a state of decomposition — yet it was with very great difficulty that we could bring the head of the bone down; and when we did so, we carried away part of the capsular ligament, and if the *pyriformis* had not been already torn, it is very probable that it would have been torn now. But when we *adducted, flexed, and carried the limb up over the pelvis*, as has been stated, the reduction was effected with the utmost ease. We varied and repeated our experiments on both joints, as often as the subject would admit, and always with the same results. I was here enabled to correct one error which I had committed in operating. If we carried the knee above the *umbilicus*, and pressed the thigh down close to the body, on a line with the side, the knee pointing towards the axilla, as I had always done, we brought the great tendon of the *gluteus maximus* into strong tension, which would compress the great trochanter so hard, that it prevented the head from mounting over the edge of the acetabulum. The reduction was effected much easier by carrying the knee and thigh about as high as the umbilicus, then abducting and rotating the thigh.

To Dr. Moore, who so kindly offered me the opportunity to demonstrate the correctness of both my theory and practice, I am much indebted and obliged.

From the foregoing facts and observations, gentlemen, I deduce the following propositions:

1. The chief impediment in the reduction of dislocations, is the indirect action of the muscles that are put upon the *stretch* by the mal-position of the dislocated bone, and not by the *contraction* of the muscles that are shortened.

2. That muscles are capable of so little extension, without hazard of rupture, beyond their normal length, that no attempt should be made to stretch them further, in order to reduce a dislocation, if it can possibly be avoided.

3. The general rule for reducing all luxations should be, that the limb or bone should be carried, moved, flexed or drawn, in that direction which will relax the distended muscles.

4. Dislocation of the hip on the *dorsum ilii*, an accident so serious to the patient, and so formidable to all surgeons, is reduced with the greatest ease, in a few minutes, without much pain, without an assistant, without pulleys, without "Jarvis' Adjuster," or any other mechanical means, simply by flexing the leg upon the thigh, carrying the thigh over the sound one, upward over the pelvis, as high as the umbilicus, and then by *abducting* and rotating it.

## Figures and Tables

**Figure f1:**