# Errata

**Published:** 1851-08

**Authors:** 


*Errata.*—Several important typographical errors in the valuable article on Dislocation of the Femur, contained in this No., have been pointed out by the author since the article was printed, which the reader is requested to correct.

Page 131, *sixth* line, for "a great power," read *so* great power.

Page 132, *first* line, for form read *force; seventh* line, for abductor read *adductor; twenty-sixth* line, for abductor read *adductor.*

Page 133, *fifth* line, for abductor read *adductor; eleventh* line, for expanded read *expended; thirty-fourth* line, for abductor read *adductor; thirty-fifth* line, for treated read *stretched.*

Page 136, *twenty-first* line, for pelvis read *pulley; thirty-fifth* line, for slided read *slides.*

